# Reduced rate of force development under fatigued conditions is associated to the decline in force complexity in adult males

**DOI:** 10.1007/s00421-024-05561-9

**Published:** 2024-07-24

**Authors:** Samuel D’Emanuele, Gennaro Boccia, Luca Angius, Oliver Hayman, Stuart Goodall, Federico Schena, Cantor Tarperi

**Affiliations:** 1 Department of Neuroscience, Biomedicine and Movement Sciences, University of Verona, Verona, Italy; 2 Department of Clinical and Biological Sciences, University of Turin, Turin, Italy; 3 Department of Sport, Exercise, and Rehabilitation, Northumbria University, Newcastle upon Tyne, UK

**Keywords:** Entropy, Force complexity, Rapid force, Steadiness

## Abstract

**Purpose:**

This study aimed to verify whether the slowing of muscle contraction quickness, typically observed in states of fatigue, may worsen force control by decreasing the rate with which force fluctuations are modulated. Therefore, we investigated the relationship between rate of force development (RFD), and force fluctuations' magnitude (Coefficient of variation, CoV) and complexity (Approximate Entropy, ApEn; Detrended fluctuation analysis, DFAα).

**Methods:**

Fourteen participants performed intermittent ballistic isometric contractions of the plantar dorsiflexors at 70% of maximal voluntary force until task failure (under 60% twice).

**Results:**

Indices of RFD (RFD_peak_, RFD_50_, RFD_100_, and RFD_150_) decreased over time by approximately 46, 32, 44, and 39%, respectively (p all ≤ 0.007). DFAα increased by 10% (p < 0.001), and CoV increased by 15% (p < 0.001), indicating decreased force complexity along with increased force fluctuations, respectively. ApEn decreased by just over a quarter (28%, p < 0.001). The linear hierarchical models showed negative associations between RFD_peak_ and DFAα (β =  − 3.6 10^–4^, p < 0.001), CoV (β =  − 1.8 10^–3^, p < 0.001), while ApEn showed a positive association (β = 8.2 × 10^–5^, p < 0.001).

**Conclusion:**

The results suggest that exercise-induced reductions in contraction speed, lead to smoother force complexity and diminished force control due to slower adjustments around the target force. The fatigued state resulted in worsened force producing capacity and overall force control.

## Introduction

Exercise-induced fatigability can be defined as a reduction in functional capacity (Millet et al. 2023), underpinned by a plethora of physiological and psychological processes. The magnitude of performance fatigability depends on the relevant muscles' contractile ability and the nervous system's capacity to supply an appropriate activation signal generated from descending orders and sensory input for the assigned task (Enoka and Duchateau 2008). A consequence of fatigued muscle is the reduced ability to produce both maximal force, commonly assessed as a decline in maximal voluntary force (MVF), and the ability to quickly reach maximal and submaximal forces (D'Emanuele et al. 2021; Boccia et al. 2018). The capacity to maintain submaximal force production is crucial for everyday tasks and constitutes an essential element of motor control (Enoka and Duchateau 2008). Notably, muscle force output exhibits fluctuations around the required target force (Slifkin and Newell 1999), indicating that control of force is not perfectly accurate (Farina et al. 2016). The fluctuations in force during steady submaximal contractions includes oscillations at both high and low frequencies, those at high frequencies induce more frequent changes in limb acceleration, while low-frequency oscillations lead to gradual changes in limb acceleration (Yacoubi and Christou 2024). Low frequency oscillations are typically attributable to the low-frequency oscillations in the discharge rates of the activated motor units (Negro et al. 2009). Muscle fatigability affects these fluctuations by increasing the magnitude (Hunter and Enoka 2001) and decreasing the complexity (Pethick et al. 2015, 2019b) thus demonstrating a poorer ability to control force in the presence of muscle fatigue (Slifkin and Newell 1999; Enoka et al. 2003). The reduction in complexity during isometric contractions has been showed to diminish with fatiguing tasks performed at maximal and submaximal intermittent or sustained contractions (Pethick et al. , 2015, 2019a, b). These findings suggest that force production becomes smoother, more consistent, and more conceivable as fatigability progresses. A decrease in torque complexity indicates a decline in motor control, with experimental evidence suggesting to be influenced by central and peripheral processes that modify excitatory and inhibitory properties of the motor unit pool (Kilner et al. 1999; Pethick et al. 2015), and consequently the ability to adjust motor output in reaction to external perturbations. Measures of complexity are derived from non-linear dynamics and information theory. These measures, such as approximate entropy (ApEn; Pincus 2001) and detrended fluctuation analysis (DFAα; Peng et al. 2009), assess the randomness or regularity of a system's output and is fractally scaled over time. Various factors, including muscle testing (Clark et al. 2023), joint angle (Pethick et al. 2021b), age (Pethick et al. 2022), training status (Tracy 2007), and discharge rate of motor unit activation (Moritz et al. 2005), can affect the amplitude of force fluctuations. Taken together, this information showed that variability in maximal contractile ballistic force is determined by neural activation and interaction with peripheral factors (Del Vecchio et al. 2019; Raffalt et al. 2023).

Although MVF is the most investigated parameter, in many sporting scenarios and activities of daily living, the time available to apply force (typically <200 ms; Maffiuletti et al. 2016) is longer than the time required to develop maximal force (>300 ms). Therefore, the rate of force development (RFD) is considered functionally more important than the capacity to produce maximal force as it reflects the ability to rapidly increase muscle force after the onset of a quick voluntary contraction (Maffiuletti et al. 2016). Moreover, RFD magnitude is an essential factor for completing and optimising rapid movements. For example, in movements that involve leaving the ground (i.e., jumping), it is crucial to generate large ground reaction forces in a short time, which translate to a large magnitude of RFD (Duchateau and Amiridis 2023). Notably, RFD is affected by muscle fatigability even more than MVF (D'Emanuele et al. 2021). For example, Viitasalo and Komi (1981) found that 100 ballistic contractions with 3 s of holding phase of the knee extensor muscles decreased RFD more than MVF (36 and 24%, respectively). The same trend is supported by (Boccia et al. 2023) through 100 burst-like ballistic voluntary contractions (≈150 ms), with a greater decrease in RFD at 50 and 100 ms (−17.9 and 8.9%) from the onset than MVF (−5.8%) justifying it as a decrement in the net neural drive. RFD is influenced by various central and peripheral factors within the neuromuscular system (Maffiuletti et al. 2016): the muscle excitation seems to be prominent in the early phase of contraction (≤50 ms from the onset), while contractile characteristics are more relevant for late phase (≥100 ms from the onset; D'Emanuele et al. 2023; Folland et al. 2014; Del Vecchio et al. 2018). Consequently, the decrease in early RFD tends to be more related to a decrease in muscle excitation (Buckthorpe et al. 2014; Boccia et al. 2023), while a decrease in late RFD tends to be more related to a decrease in contractile capacity (Varesco et al. 2022).

The multi-scale anatomical structures and processes that determine the magnitude of RFD during rapid contraction are partially shared with the ones that determine the control in force during steady contractions. The slowing of the rate of force production typically seen in fatigued conditions may worsen the force control by decreasing the speed with which the force fluctuations are modulated. For example, Pethink and colleagues found a decrease in force complexity, thus a reduction in force control, only when there was a clear decrease in contractile capacity (Pethick et al. 2016). Nevertheless, this is not sufficient to explain the increase in low-frequency oscillations, which is typically due to the increased strength of common synaptic input to motor neurons during fatiguing contractions (Castronovo et al. 2015). Not only does the rate of change in force play an important role within sensorimotor feedback system (Lin et al. 2019). A variety of somatosensory afferent firing rates in response to stimuli have been characterised based on the rates of change in force or mechanical stress, such as cutaneous sensors (Grigg and Del Prete 2002), Golgi tendon organs (Jami et al. 1985), and muscle spindles (Blum et al. 2020). Together, it is reasonable to hypothesise that a decline in voluntary RFD would at least partially cause a worsening in the control of force production.

Accordingly, the present study aimed to evaluate the influence of intermittent, submaximal contractions performed to task failure with the dorsiflexor muscles on the rate of force development and force control metrics. We hypothesised that muscle fatigue would induce a decline in RFD and an impairment in force control. Furthermore, our novel hypothesis, was that the decline in RFD would be associated with the decline in force steadiness and complexity. We adopted a protocol based on intermittent isometric contractions because they represent a useful model with which to elicit neuromuscular fatigability (Pethick and Tallent 2022; Pethick et al. 2016). The intermittent protocols have the advantage of measuring RFD and muscle force control in the same contraction, thus allowing the development of neuromuscular fatigability-induced changes in force control to be tracked despite the lack of ecological validity (Pethick and Tallent 2022).

## Material & methods

### Participants

A convenience sample of 14 healthy male adults were recruited to participant in this study (mean ± SD; 32 ± 6 years; 77 ± 14 kg; 1.76 ± 0.10 m; BMI 25 ± 3). Participants were physically active, participating in exercise related activity at least twice a week. Exclusion criteria were any previous history of neuromuscular disorders or lower limb injury in the previous six months. All the participants were informed about the testing procedure and provided written informed consent.

### General overview

The experimental session was composed of a series of isometric contractions of the ankle dorsiflexors, and it was divided into two parts. The first part (which lasted about 15 min) constituted a warm-up and familiarisation with real-time visual feedback, which continued until the participant was able to perform the ballistic contractions without countermovement. The second part started 5 min after the first and was constituted of (1) two maximal voluntary contractions (MVCs) interspaced by 2 min; (2) the fatiguing task, and (3) another MVC immediately after the fatiguing task. Data are only presented from the second (post-familiarisation) part.

### Setup

The participants were seated with their dominant foot inserted in the dynamometer (NEG1, OTBioelettronica, Turin, Italy) with the trunk reclined to 90° (180° = supine position), the knees extended at 180°, and the dominant ankle at 100° of plantar flexion (90° = neutral position). The foot was strapped in via a metal plate attached to the level arm with the talus and phalange Velcro strapped. Participants then performed a warm-up consisting of two isometric ankle dorsiflexion at various intensities (30, 50, and 80% of perceived maximal effort). Subsequently, participants performed two 5‐s MVCs interspersed by 2 min of rest. The highest force reached in the two contractions was considered the MVF of plantar dorsiflexors (PD). Participants were instructed with standardised verbal encouragement (Sahaly et al. 2001) to contract "as hard as possible" during the MVF assessment and "as fast as possible" during the fatiguing task.

### Fatiguing task

**Fig. 1:**
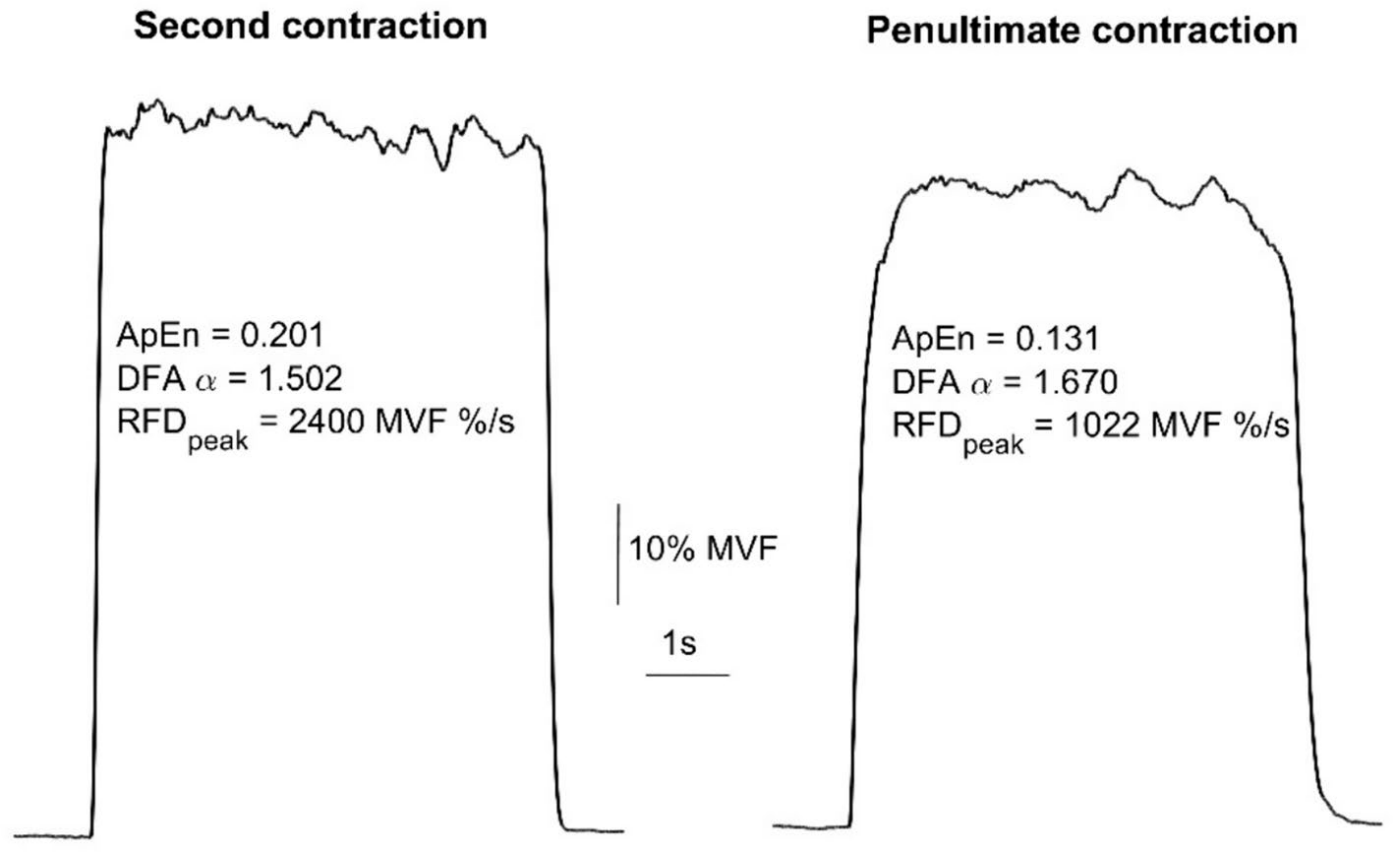
Example of force signals acquired from a single subject at the beginning (second contraction, left panel) and at the end (penultimate contraction, right panel) of the fatiguing task. The estimates of Detrended Fluctuation Analysis (DFAα), Approximate Entropy (ApEn); Rate of Force Development (RFD_peak_) are reported for each contraction

The fatiguing protocol consisted of a series of ballistic contractions with a holding phase of 6 s at 70% of MVF, interspersed by 4 s of rest (Pethick et al. 2015), until task failure. The participants were instructed to reach the target force as quickly as possible and then to maintain the target force as stably as possible. The rationale for the target force was chosen because to achieve a truly maximal RFD the target force should be at least 70% of MVF. Reaching lower levels would result in a too-slow contraction. Furthermore, to ensure a maximal speed of force production, the participants were allowed to overshoot the target force at the beginning of the contraction and then return to the target force afterwards. Task failure was decreased when participants could no longer maintain the force within a range of 10% in any phase of the contraction for two consecutive contractions, including the overshoot. A display screen was positioned in front of the participants to provide real-time visual—feedback on the force production. A representative example of force trace is reported in Fig. 1.

### Data analysis

During offline analysis, the analog force signal was converted to newtons (N) and low-pass filtered (4th order, zero-lag, Butterworth, cut-off frequency 1000 Hz). Contractions that showed any countermovement (i.e., a negative shift > 0.5 N in the preceding 200 ms) were excluded, and the onset was visually selected by the same researcher (Tillin et al. 2010). The baseline noise of the force signal was on average 0.12% of MVF. RFD at 50, 100, and 150 ms (RFD_50_, RFD_100_, RFD_150_) from the onset of each contraction (see (D'Emanuele et al. 2023) for details) were calculated and normalised to the MVF. RFD_peak_ was also calculated as the highest first derivative of the force signal, adopting a moving window of 20 ms.

All measures of variability and complexity were calculated visually selecting the 5 s of the steadiest part of each contraction, avoiding including overshooting that some subjects did at the beginning of the contraction to search for the target. The amplitude of variability in the torque output of each contraction was measured using the coefficient of variations (CoV) that quantifies the magnitude of fluctuations normalised to the mean force output. ApEn (Pincus 1991) was used to estimate the complexity of force output and quantify the negative natural logarithm of the conditional probability that a template of length *m* is repeated during a time series. *m* (pattern length) was set at 2, and *r* (tolerance) was set at 0.1 of the standard deviation of the isometric force during the steady state of the contractions (Pethick et al. 2015). DFAα is a method to quantify the correlations in a time series and to identify long-term dependencies or trends within the data. The fluctuation exponent obtained from DFAα, according to Pethick et al. (2015), reflects the presence of long-term correlations in the signal. In detail, the DFAα exponent theoretically ranges approximately from ~ 0.5 to ~ 1.5 and distinguishes between outputs that exhibit randomness (e.g. white noise, α = 0.5), statistically self-similar fluctuations (e.g. pink or 1/f noise, α = 1.0), or Brownian characteristics (e.g. with long-term memory, α = 1.5). The data were analysed with a customised script (MatLab ver. 2023b, TheMathWorks Inc., Natick, MA, Massachusetts, USA).

### Statistical analysis

Values are reported as mean ± SD. Kolmogorov–Smirnov normality test was used to assess distributions normality. The assumption of sphericity was checked by means of Mauchly's test. When the assumption of sphericity was violated, a Greenhouse–Geisser correction was applied. The effect size in ANOVA was reported as η^2^. Threshold values for η^2^ were: = 0.01, small; = 0.06, medium; = 0.14 large. Post hoc comparisons were corrected using the Bonferroni method. Pre vs. post-comparison of MVF values was analysed with a t-test for paired samples. To test the time course of mechanical variables along the fatiguing task, the length of the fatiguing task of each subject was divided into five equal blocks of contractions to obtain five-time points (Q1, Q2, Q3, Q4, and Q5). The estimates of each mechanical variable (RFD, steadiness, and complexity) were averaged within the five blocks. Then, the five-time points were compared with a repeated measures ANOVA to test the relationship between RFD_peak_ and the estimates of steadiness and complexity. We performed a multilevel mixed linear regression analysis using the RFD, steadiness, and complexity estimates of all available contractions. Subjects were included as random effects (e.g. *DFAα ≈ RFD*_*peak*_ + *[1|subjects]).* The level of statistical significance was set to p = 0.05. Descriptive statistics and ANOVA were performed using JASP software (v0.17.1), while the multilevel linear regression analysis were performed in R (v4.1.1, R Core Team, Vienna, Austria, 2021) through the package lme4 (v1.1.19).

## Results

**Fig. 2:**
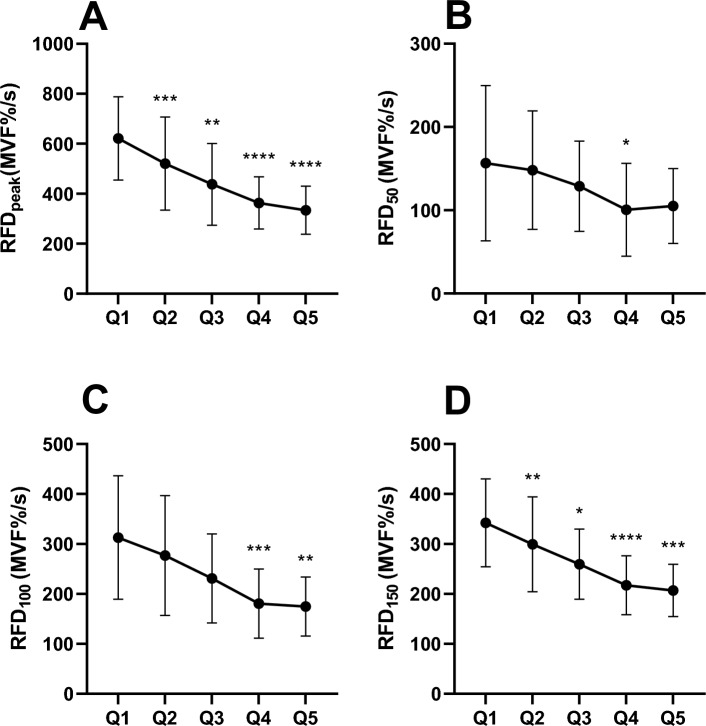
The four panels represent the rate of force development (RFD) values assessed at peak (RFD_peak_; panel **A**), at 50 ms from the contraction's onset (RFD_50_; panel **B**), at 100 ms from the contraction's onset (RFD_100_; panel **C**), at 150 ms from the contraction's onset (RFD1_50_; panel **D**). The number of contractions was divided into 5 equally distributed blocks for each subject. Each Q represents the mean ± standard deviations of each of these blocks across all subjects normalised for maximal voluntary force (MVF) at PRE. *p = 0.05; **p ≤ 0.01; ***p ≤ 0.001; ****p ≤ 0.0001

Subjects performed an average of 24 ± 11 contractions (≈4 min). Pre-exercise MVF was 386 ± 44 N, while immediately after the task failure, it was reduced to 244 ± 41 N (Δ −142 ± 25 N; −37%; p < 0.001). RFD_peak_ (normalised for MVF at PRE) decreased by 46% from 621 ± 167 to 334 ± 165%MVF/s (F_4,44_ = 36.1; p < 0.001; η^2^ = 0.766). RFD_50_ decreased by 32% from 156 ± 93 to 105 ± 44%MVF/s (F_4,44_ = 4.0; p = 0.007; η^2^ = 0.267). RFD_100_ decreased by 44% from 312 ± 116 to 174 ± 85%MVF/s (F_4,44_ = 15.2; p < 0.001; η^2^ = 0.581). RFD_150_ decreased by 39% from 342 ± 83 to 207 ± 75%MVC/s (F_4,44_ = 27.9; p < 0.001; η^2^ = 0.717). The time course of RFD estimates is reported in Fig. 2.

**Fig. 3:**
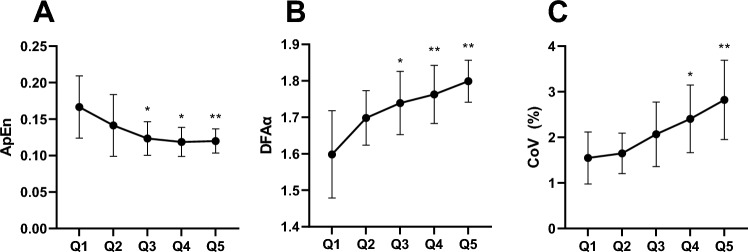
**A** Approximate Entropy (ApEn) significantly decreased over the time-period; **B** Detrended Fluctuation Analysis (DFAα) significantly increased over the time-period; **C** Coefficient of Variation (CoV) significantly increased over the time period. The number of contractions was divided into five equally distributed blocks for each subject. Each Q represents the mean ± standard deviations of each of these blocks across all subjects. *p = 0.05; **p ≤ 0.01

ApEn decreased by 28% from 0.167 ± 0.043 to 0.120 ± 0.016 (F_4, 44_ = 7,6; p < 0.001; η^2^ = 0.409; 3A) whereas DFAα increased by 10% from 1.60 ± 0.17 to 1.80 ± 0.08 (F_4,44_ = 14.8; p < 0.001; η^2^ = 0.575; Fig. 3B). CoV increased by 15% from 1.5 ± 0.5 to 2.8 ± 0.8% (F_4,44_ = 9.6; p < 0.001; η^2^ = 0.468; Fig. 3C). The time course of steadiness and complexity estimates are reported in Fig. 3.

**Fig. 4:**
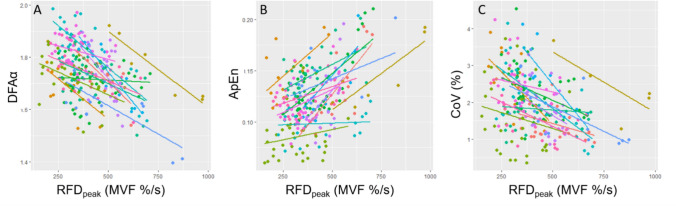
Multilevel linear regression analysis were applied to explore if, within the subject, the peak of the rate of force development (RFD_peak_) influences **A** Detrended Fluctuation Analysis (DFAα), **B** Approximate Entropy (ApEn); **C** coefficient of variation (CoV) of force. Each dot represents a contraction, each line represents a participant

Multilevel mixed linear regression analysis showed a negative influence of RFD_peak_ on DFAα (F_1,314_ = 73.6, β =  − 3.6 10^–4^, SE = 4.2 10^–5^, p =  < 10^–16^, see Fig. 4A), a positive influence on ApEn (F_1,339_ = 28.6, β = 8.2 10^–5^, SE = 1.5 10^–5^, p =  < 10^–7^, see Fig. 4B) and a negative influence on CoV (F_1,275_ = 22.6, β =  − 1.8 10^–3^, SE = 3.8 10^–4^, p =  < 10^–6^, see Fig. 4C).

## Discussion

In this study, we analysed the relationship between RFD, force steadiness and complexity during an intermittent fatiguing task of the ankle dorsiflexors'. Our initial hypothesis has been confirmed, as muscle fatigability achieved through the task led to a decline in RFD (≈ − 40%), along with a deterioration in force complexity and steadiness. Furthermore, the results have, for the first time, confirmed our second hypothesis. Indeed, we report that RFD is correlated with force steadiness and complexity, and we provided interpretation for these results despite not being able to discuss a causal relationship among the data.

Following an intermittent dorsiflexor task, we found a decrease in the speed at which participants could contract their plantar dorsiflexors. Specifically, we found a considerable reduction in RFD_peak_, RFD_50_, RFD_100_ and RFD_150_ (−46, −32, −44, and −39%, respectively). Previous work has investigated the effect of intermittent tasks in different muscle groups. Buckthorpe et al. (2014) showed that following intermittent maximal knee extensors contractions, the early force production (≤50 ms) largely decreased. These results were influenced by both contractile and neural factors with a predominant role of central mechanisms (Buckthorpe et al. 2014). Kennedy et al. (2011) showed that following a fatiguing bi-pedal dorsiflexor task at 70% MVF, contractile factors were mainly affected, whilst central mechanisms (i.e., voluntary activation) remained unchanged post-exercise.

The present fatiguing task also affected the steadiness and complexity of the force signal. Specifically, ApEn, DFAα, and CoV showed a –28, +10 and +15% change, respectively, all categorised as large effects. These findings are in line with previous work (Pethick et al. 2015, 2018, 2019b) investigating sustained and intermittent contractions. Indeed, Pethick et al. (2015) showed a decrease in ApEn (from 0.65 to 0.27), while the DFAα increased (from 1.35 to 1.55) at the end of an intermittent submaximal fatiguing task. Collectively, these results show that fatigability was associated with reduced complexity in motor output when the muscle is driven sub-maximally. A reduction in complexity diminishes these degrees of freedom (Peng et al. 2009; Stergiou and Decker 2011), leading to system components becoming less responsive to inputs over time (Pethick et al. 2015). This complexity reduction seems to be associated with alterations in motor control, movement mechanics, and coordination, which are speculated to heighten the likelihood of unsuccessful execution of motor tasks. The decrease in steadiness and the consequent change in CoV can be attributed to the characteristics of the control signal produced by the central nervous system (CNS), resulting in increased oscillations of synaptic inputs common to motor units (Pereira et al. 2019). The differences in strength variability could be related to the CNS's ability to drive low frequency osclillation toward the muscle (Dideriksen et al. 2012). However, the change in force complexity is not wholly due to factors within the nervous system but is affected by contractile factors Raffalt et al. (2023). The translation of the information embedded in the neural signal into actual movements relies on the mechanical properties of the muscle–tendon unit. Indeed, Raffalt et al. (2023) showed that the temporal information in the force signal after 2 min of isometric dorsiflexion at 10% of MVF did not reflect that of the underlying neural signals as measured by EEG. In the present study, reduced muscle contractility, which is likely to occur in a fatigued state (Bigland-Ritchie et al. 1986) probably led to alterations in force steadiness.

For the first time, we provide data on the interrelation between two fundamental characteristics of force production, speed and steadiness. Adopting a hierarchical linear regression we provided the most appropriate linear fit for each participant, allowing us to detect associations between variables that might otherwise be obscured due to aggregation of non-independent values (Fig. 4). Using such a within-subject approach, we found that the fatigability-induced decrease in RFD_peak_ is at least partially associated with a decrease in force steadiness (Fig. 4C). Even more interestingly, the decrease in RFD is associated with a loss in force complexity (Fig. 4A) and an increased predictability (Fig. 4B). That RFD is inversely correlated to DFAα, suggests that the speed of contraction decrease in the presence of fatigue, leads to the force signal becoming more Brownian in nature, with a smoother, long-term memory. In other words, if the neuromuscular system can control the force with less quickness, more long-term oscillations might occur. Similarly, the positive relationship between RFD_peak_ and ApEn might show that the decrease in contraction speed likely caused a slowing in force production adjustments, thus leading to a slower, more predictable fluctuation in force. We can conclude that the slower and less precise motor control commonly seen in fatigued conditions are, therefore, two interrelated phenomena despite the fact that we are unable to disentangle which physiological phenomena can explain this relationship.

### Limits and future perspectives

The interpretation and generalization of these findings are limited by the fact that only male subjects were included. This must be recognized as a weakness of the present study. Another weakness is that we adopted linear models to establish the relationships between RFD and force steadiness measures, but those relationships might not follow a linear trend. The measures of complexity during isometric muscle contractions can depend on the muscle tested, level of activation, and level of training, which are all factors that should be controlled in this type of work (Tracy 2007; Pethick et al. 2021b).

Future studies should aim to investigate force fluctuations and its neuromuscular determinants on different muscles, tasks, and contraction intensities. Utilising a greater array of methods will lead to a more complete understanding of force variability (Pethick et al. 2021a), and the combination of different techniques will provide a greater insight into the central and peripheral factors associated with both RFD and force complexity changes with fatigability.

## Conclusions

This study confirmed that neuromuscular fatigability was associated with reduced complexity in motor output, assessed through DFAα and ApEn, during repeated isometric intermittent contractions of the dorsiflexors. Moreover, the slowing of force production capacities, as evidenced by the RFD decline, seems to be associated with changes in complexity because it lessens the rapidity of adjustments around the target force.

## Data Availability

All data are available from the corresponding author upon reasonable request.

## Abbreviations

ApEn: Approximate entropy
CoV: Coefficient of variations
DFAα: Detrended fluctuation analysis
MVC: Maximal voluntary contraction
MVF: Maximal voluntary force
RFD: Rate of force development
